# Cystatin C for predicting all-cause mortality and rehospitalization in patients with heart failure: a meta-analysis

**DOI:** 10.1042/BSR20181761

**Published:** 2019-02-05

**Authors:** Shenghua Chen, Yangzhang Tang, Xueyin Zhou

**Affiliations:** 1 Health Service Centre of West Street Coummunity of Xiangshan District, Huaibei 235000, Anhui Province, China; 2 Department of Internal Cardiovascular Medicine of the People’s Hospital, Huaibei 235000, Anhui Province, China

**Keywords:** all-cause mortality, cystatin C, heart failure, meta-analysis

## Abstract

Circulating cystatin C (cys-C/CYC) has been identified as an independent predictor of all-cause mortality in patients with coronary artery disease and the general population. This meta-analysis aimed to systematically evaluate the association between elevated cys-C level and all-cause mortality and rehospitalization risk amongst patients with heart failure (HF). PubMed and Embase databases were searched until December 2017. All prospective observational studies that reported a multivariate-adjusted risk estimate of all-cause mortality and/or rehospitalization for the highest compared with lowest cys-C level in HF patients were included. Ten prospective studies involving 3155 HF patients were included. Meta-analysis indicated that the highest compared with lowest cys-C level was associated with an increased risk of all-cause mortality (hazard ratio (HR): 2.33; 95% confidence intervals (CI): 1.67–3.27; *I^2^* = 75.0%, *P*<0.001) and combination of mortality/rehospitalization (HR: 2.06; 95%CI: 1.58–2.69; *I^2^* = 41.6%, *P*=0.181). Results of stratified analysis indicated that the all-cause mortality risk was consistently found in the follow-up duration, cys-C cut-off value or type of HF subgroup. Elevated cys-C level is possibly associated with an increased risk of all-cause mortality and rehospitalization in HF patients. This increased risk is probably independent of creatinine or estimated glomerular filtration rate (eGFR).

## Introduction

Heart failure (HF) is a global healthcare burden with unacceptable risk of morbidity, rehospitalization, and mortality [1,2]. According to the different pathophysiological mechanisms, patients are commonly classified into HF with preserved ejection fraction (EF) (HFpEF) or HF with reduced EF (HFrEF). HFpEF is associated with poor clinical outcomes and severe cardiovascular dysfunction [3,4]. Frequent hospitalization and prolonged hospital stay were the typical features of patients with HF [5,6]. HFpEF patients had a higher incidence of non-HF hospitalizations, while HFrEF patients had a higher incidence of HF hospitalizations [7]. Therefore, early prognostic stratification of HF patients at high risk of mortality and rehospitalization is in urgent need.

Biomarkers are frequently used to predict adverse outcomes in patients with HF [8]. HF may lead to renal dysfunction and cardiorenal syndrome through low cardiac output, accelerated atherosclerosis, inflammation, and increased venous pressure [9]. Renal function offers valuable information for the prognostic classification of patients with stable or decompensated HF [10]. HF patients with renal impairment represent a high-risk group with an approximately 50% greater risk of mortality than those with normal renal function [11]. Use of serum creatinine and creatinine-based formula to quantitate renal function is routinely applied in clinical practice. However, age, gender, muscle mass, physical activity, or diet can influence creatinine level [12].

Cystatin C (cys-C) is a small protein molecule producing by virtually all nucleated cells in the human body. Circulating cys-C level has been introduced as a more sensitive biomarker of early renal impairment [13], particularly in those with a normal creatinine level [14]. Several studies reported the association between cys-C level and all-cause mortality/rehospitalization risk amongst HF patients. However, no previous meta-analysis has been conducted to systematically evaluate the prognostic significance of cys-C level in HF patients. Nevertheless, the magnitude of the prognostic value varied considerably across studies.

In the current study, we performed a meta-analysis of prospective studies to qualitatively evaluate the prognostic value of cys-C level amongst HF patients in terms of all-cause mortality and/or rehospitalization.

## Methods

### Search strategy

We conducted and reported this meta-analysis in accordance with the checklists of the Preferred Reporting Items for Systematic Reviews and Meta-Analyses (PRISMA) guidelines [15]. Ethical approval is unnecessary as the present study does not contain individual patients data. PubMed and Embase databases were searched until December 2017 using the following keywords: ‘cystatin C’ AND ‘heart failure’ AND ‘mortality’ OR ‘death’ AND ‘prospective’ OR ‘follow-up’. No language restriction was applied. In addition, we also manually searched the reference lists of relevant articles to identify any additional studies.

### Study selection

The full-text articles meeting the following inclusion criteria were selected: (i) prospective observational studies enrolling the HF patients; (ii) baseline circulating cys-C level as exposure; (iii) outcome measures were all-cause mortality and/or rehospitalization; and (iv) provided multivariate-adjusted risk estimate of all-cause mortality and/or rehospitalization for the highest compared with the lowest category of cys-C level. Exclusion criteria were: (i) study design was a retrospective or conference abstract; (ii) participants were not HF patients; and (iii) multiple articles from the same study population.

### Data extraction and quality assessment

Two authors independently extracted data from included studies. Data extracted included the first author’s last name, publication year, country of origin, study design, patients’ number, age, proportion of men, comparison of cys-C cut-off value, outcome measure, event number, most fully adjusted hazard ratio (HR) or risk ratio (RR) and 95% confidence intervals (CI), follow-up period, and adjusted covariates. Any differences between the two authors were resolved by consultation with a third author. The methodological quality of the included studies was assessed by a 9-star Newcastle–Ottawa Scale (NOS) for cohort studies [16]. Based on this scale, studies with a rating of >7 stars were grouped as good quality.

### Statistical analyses

All the meta-analyses were performed using STATA version 12.0 (StataCorp, TX, U.S.A.). We pooled the HR with 95% CI for the highest compared with the lowest cys-C level. Heterogeneity was evaluated using the *I^2^* statistic and Cochrane *Q* test. The *I^2^* statistic > 50% or *P*<0.10 of the Cochran *Q* test was considered the presence of significant heterogeneity. We chose a random-effect model when significant heterogeneity was present; otherwise, a fixed-effect model was applied. Subgroup analyses were performed according to region (Europe compared with others), follow-up duration (>1.5 compared with <1.5 years), sample sizes (≥400 compared with <400), type of HF (acute compared with chronic), and cys-C cut-off value. Sensitivity analysis was conducted by sequentially removing any one study at each turn to observe the reliability of the pooled risk estimate. In addition, we assessed the publication bias using the Egger’s linear regression test [17] and Begg’s rank correlation [18].

## Results

### Literature search and study characteristics

**Figure 1:**
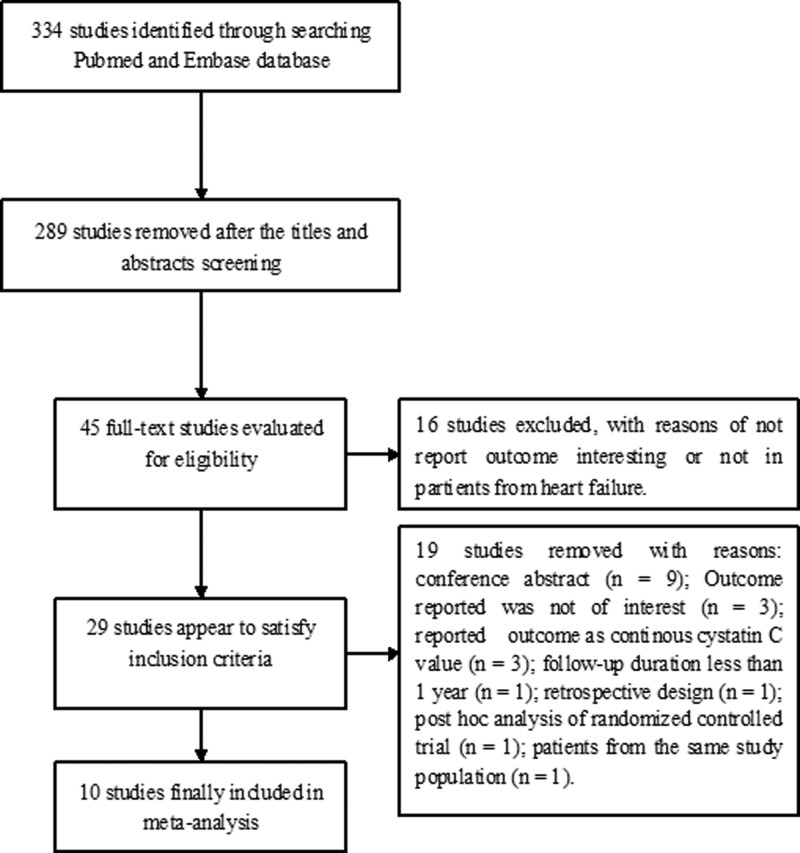
Flow chart of the study selection process

Figure 1 describes the flow chart of the study selection process. In brief, a total of 344 potentially relevant articles were identified in our initial literature search. After screening the titles and abstracts, 289 articles were excluded. The remaining 45 articles were determined for a detailed evaluation. Thirty-five articles were further removed mainly because they did not report the outcome of interest, conference abstract or reported outcome as continuous cys-C value. Thus, ten studies [19–28] were finally included in this meta-analysis.

Table 1 summarizes the main characteristics of the included studies. Sample sizes ranged between 162 and 2278 in individual studies. Seven studies [20,22–25,27,28] were conducted in Europe, two studies [19,21] in U.S.A., and one study [26] in China. Follow-up duration ranged from 12 months to 6.5 years. Different cut-off levels were used for defining cys-C elevation. Overall methodological quality of the included studies was moderate to good (ranging from 5 to 7 stars) on a 9-star NOS.

**Table 1:** Summary of clinical studies included in meta-analysis

Author (year)	Country	Study design	Patients (% male)	Age (years)	Baseline LVEF (%)	Comparison of CYC (mg/l)	Outcome measures HR or RR (95% CI)	Follow-up duration	Adjustment for covariates	Overall NOS
Shlipak et al. (2005) [19]	U.S.A.	Prospective cohort study	Chronic HF 279 (49.5)	76.6 ± 6	NP	Quartile 4 compared with1; ≥1.56 compared with ≤1.03	Total deaths:182	6.5 years	Age, gender, BMI, stroke, cancer, hypertension, anemia, and lipid-lowering medication	7
							2.15 (1.30–3.54)			
Lassus et al. (2007) [20]	Finland	Prospective study	AHF 480 (50.0)	74.8 ± 10.4	45 ± 16	>1.3 compared with <1.3	Total deaths: 122	1.0 year	Age, gender, SBP, DBP, hyponatremia, anemia, creatinine, and NT-proBNP	6
							3.2 (2.0–5.3)			
Campbell et al. (2009) [21]	U.S.A.	Prospective cohort study	AHF 240 (50.0)	63 ± 14	35 ± 20	Quartile 4 compared with 1–3	Total deaths: 53	1.0 year	Age, race, gender, type of HF, QRS duration, LVRF, cancer, cirrhosis, and DM	6
							2.0 (1.03–3.88);			
							Death/rehospitalization: 153;			
							1.94 (1.27–2.95)			
Manzano-Fernández et al. (2011) [22]	Spain	Prospective study	AHF 220 (54)	72.2 ± 11.9	46.4 ± 16.6	>1.05 compared with <1.05	Death/rehospitalization: 116	1.37 years	Age, NYHA, glucose, ST-segment elevation MI, leukocytes, β-trace protein, in-hospital inotrope use, creatinine, eGFR, urea nitrogen, troponin-T, and NT-proBNP	6
							1.73 (1.15–2.62)			
Carrasco-Sánchez et al. (2011) [23]	Spain	Prospective study	AHF 218 (49.9)	75.6 ± 8.7	LVEF >45%	Quartile 4 compared with 1; >2.06 compared with ≤1.12	Total deaths: 70	1.0 year	Age, creatinine, urea nitrogen, HB, NT-proBNP, hyponatremia,and NYHA	7
							8.14 (2.33–28.4);			
							Death/rehospitalization: 126; 3.40 (1.86–6.21)			
Pérez-Calvo et al. (2012) [24]	Spain	Prospective study	AHF 526 (45)	76 (70–81)	80.7% cases LVEF ≥45%	>1.25 compared with <1.25	Total deaths: 66	1.0 year	Age, gender, NT-proBNP, total cholesterol, urea, HF with preserved EF, NYHA, AF, DM and hypertension	7
							2.86 (1.72–4.77);			
Carrasco-Sánchez et al. (2014) [25]	Spain	Prospective study	AHF 195 (42.2)	76.3 ± 8.2	71.8% cases LVEF ≥45%	≥1.32 compared with <1.32	Total deaths: 40	1.0 year	Age, NT-proBNP, anemia, hyponatremia, LVEF, serum creatinine, and NYHA	7
							4.87 (1.92–12.36)			
Ruan et al. (2014) [26]	China	Prospective cohort study	AHF with AKI 162 (53.7)	51.9 ± 15.4	39.1 ± 11.6	Tertile 3 compared with 1; >1.46 compared with <1.11	Total deaths: 45	1.0 year	Multivariate logistic regression analysis	5
							2.72 (1.92–4.28)			
Jackson et al. (2016) [27]	U.K.	Prospective study	AHF 628 (58.4)	70.8 ± 10.6	40.1 ± 12.1	>1.6 compared with <1.6	Total deaths: 290	3.2 years	Age, gender, smoking, NYHA, LVEF, HR, SBP, BMI, peripheral edema, bilirubin, urate, creatinine, HB, HbA1c, lymphocyte/red cell distribution width, and BNP	7
							1.13 (0.81–1.57)			
Breidthardt et al. (2017) [28]	Switzerland	Prospective study	AHF 207 (59%)	80 (74–85)	40 (25–55)	≥1.5 compared with <1.5	Total deaths: 95	1.72 years	Early AKI, SBP, urea at presentation, creatinine, serum sodium and BNP	7
							1.41 (1.02–1.95)			

Abbreviations: AF, atrial fibrillation; AKI, acute kidney injury; BMI: body mass index; DBP, diastolic blood pressure; DM, diabetes mellitus; EF, ejection fraction; eGFR, estimated glomerular filtration rate; HB, hemoglobin; HbA, glycosylated HB; LVEF, left ventricular EF; NP, not reported; NT-proBNP, N-terminal B-type natriuretic peptide; SBP, systolic blood pressure.

### All-cause mortality

**Figure 2:**
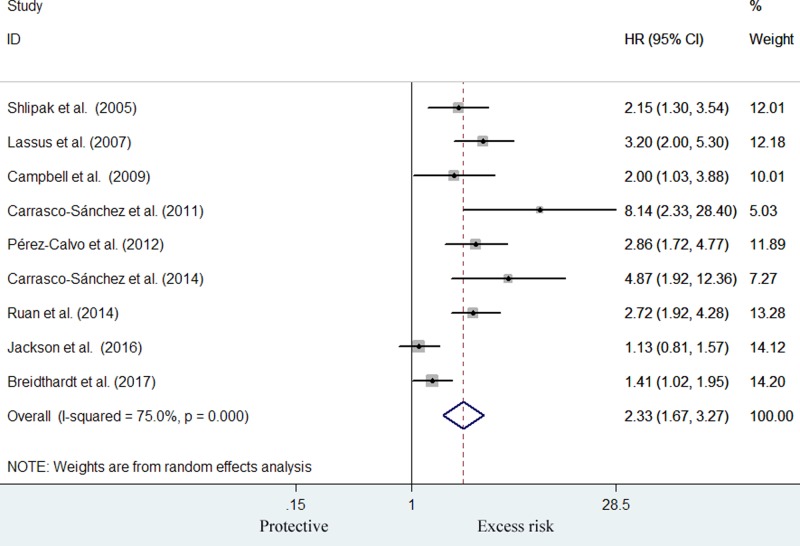
Forest plot showing HR and 95% CI of all-cause mortality for the highest compared with the lowest category of cys-C level in a random-effect model

Nine studies [19–21,23–28] reported the prognostic value of the elevated cys-C level with all-cause mortality risk. As shown in Figure 2, meta-analysis showed that cys-C level was associated with greater risk of all-cause mortality (HR: 2.33; 95% CI: 1.67–3.27) for the highest compared with lowest cys-C category in a random-effect model, with evidence of significant heterogeneity (*I^2^* = 75.0%, *P*<0.001). Sensitivity analyses showed minimal changes in magnitude of the pooled risk estimate when any one study was removed from the meta-analysis (Supplementary Table S1). Subgroup analyses suggested that prognostic values were consistently found in each named subgroup (Table 2). Publication bias was observed in the Egger’s test (*P*=0.016) but not Begg’s test (*P*=0.118). However, the result for publication bias is potentially unreliable because the number of studies analyzed was less than the recommended arbitrary minimum number of ten [29].

**Table 2:** Subgroup analyses of all-cause mortality

Subgroup	Number of studies	Pooled HR	95% CIs	Heterogeneity across studies
Sample sizes				
≥400	3	2.11	1.07–4.15	*P*<0.001; *I^2^* = 88.2%;
<400	6	2.49	1.64–3.79	*P*=0.011; *I^2^* = 66.2%
Follow-up duration				
>1.5 years	3	1.44	1.04–1.99	*P*=0.109; *I^2^* = 54.8%
<1.5 years	6	2.97	2.33–3.78	*P*=0.387; *I^2^* = 4.6%
Region				
Europe	6	2.46	1.49–4.05	*P*<0.001; *I^2^* = 82.3%
Others	3	2.39	1.80–3.17	*P*=0.654; *I^2^* = 0.0%
Cys-C cut-off value				
Single category	6	2.11	1.39–3.21	*P*<0.001; *I^2^* = 77.9%
≥3 category	3	2.82	1.76–4.53	*P*=0.151; *I^2^* = 47.1%
Types of HF				
Chronic HF	1	2.15	1.30–3.55	—
Acute HF	7	2.39	1.63–3.50	*P*<0.001; *I^2^* = 78.0%

### Combination of mortality/rehospitalization

**Figure 3:**
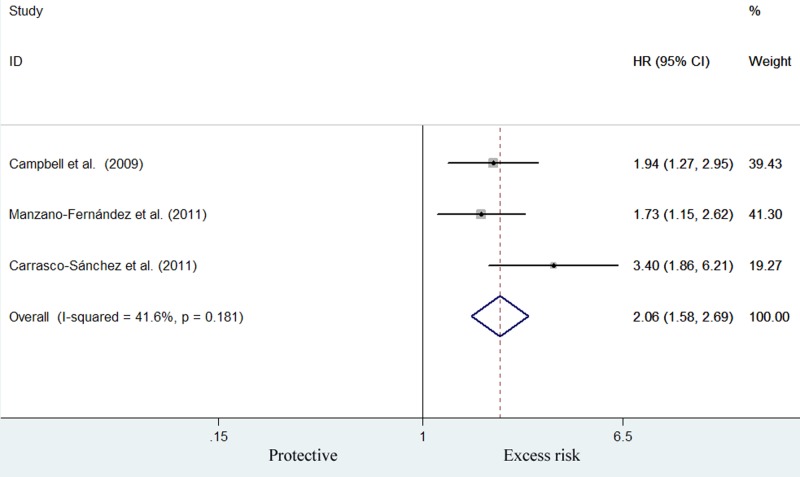
Forest plot showing HR and 95% CI of combination of mortality/rehospitalization for the highest compared with the lowest category of cys-C level in a fixed-effect model

Three studies [21–23] reported the association between the elevated cys-C level and the combination of mortality/rehospitalization risk. As shown in Figure 3, meta-analysis indicated that cys-C level was associated with higher risk of combination of mortality/rehospitalization risk (HR: 2.06; 95% CI: 1.58–2.69) for the highest compared with lowest cys-C category in a fixed-effect model, without evidence of significant heterogeneity (*I^2^* = 41.6%, *P*=0.181). Sensitivity analyses indicated that removal of any one study did not significantly change the conclusion (Supplementary Table S2).

## Discussion

This meta-analysis suggested that elevated cys-C level was possibly associated with an increased risk of all-cause mortality and rehospitalization in patients with HF. This increased risk may be independent of creatinine or urea nitrogen. HF patients with the higher category of cys-C level had a 2.3-fold greater risk of all-cause mortality. These findings are expected to improve the stratification of HF patients at higher risk of mortality and rehospitalization. This meta-analysis further reinforces the prognostic value of cys-C in patients with HF.

Subgroup analyses indicated that studies reporting single dichotomous cut-off value of cys-C level showed a similar high risk estimate, revealing even moderate increase in cys-C level could predict mortality risk of HF patients. The prognostic value of cys-C weakened with the lengthening of the follow-up period. This result may be explained that higher short-term mortality mainly reflects the worse heart function rather than renal dysfunction.

To the best of our knowledge, no previous meta-analysis has evaluated the prognostic significance of cys-C in HF patients. The finding of the current meta-analysis highlights that evaluation of renal function using cys-C should play an important role in risk stratification of this population. In clinical practice, renal function is usually detected using serum creatinine-based formula or Cockcroft–Gault estimate. However, both of them may lead to overestimation or underestimation of renal dysfunction [30]. cys-C is freely filtered by the glomerular membrane without secretion or reabsorption to the blood flow [31], so cys-C level is a sensitive marker of early kidney function [32,33]. Shlipak et al. [19] reported that cys-C level was a superior predictor of mortality than creatinine in elderly patients with chronic HF. Furthermore, higher cys-C level was associated with higher risk of all-cause mortality even in individuals with the normal estimated glomerular filtration rate (eGFR) [34,35]. These data revealed that elevated cys-C level for predicting all-cause mortality risk may be independent of serum creatinine in these populations. A previous meta-analysis [36] summarized that in the general population, individuals with the highest cys-C level had a 72% higher risk of all-cause mortality. Another well-designed meta-analysis [37] showed that elevated circulating cys-C significantly increased by 2.27-fold greater risk of all-cause mortality in people with suspected or established coronary artery disease.

A number of studies that did not satisfy our predefined inclusion criteria for this meta-analysis also evaluated the prognostic role of cys-C in HF patients. Results from the ASCEND-HF trial showed higher baseline but not follow-up cys-C level was associated with an increased risk of 30-day adverse events and 180-day all-cause mortality [38]. A retrospective study suggested that cys-C could be better prognostic biomarker for combination of recurrent HF or cardiac death when compared with N-terminal pro-B-type natriuretic peptide [39]. Measurement of cys-C level could improve early risk stratification of cardiac death in acute HF patients with normal to moderately impaired renal function [40]. Furthermore, the prognostic value of cys-C in HF patients was also supported by the continuous variable analysis (per SD increase) [41,42]. These data suggest that prognostic value of cys-C may be a superior biomarker in risk stratification of HF patients with normal or slightly impaired renal function.

Several limitations should be mentioned in this meta-analysis. First, inflammatory status, hyperthyroidism, glucocorticoids use, and current smoking status could have influenced cys-C level [43–45]. Lack of adjustment of these residual or unmeasured confounding factors may have overestimated the risk estimate. Second, cys-C level was determined only at baseline which may have caused misclassification of patients in each category of cys-C level. Follow-up monitoring of cys-C level may be more accurate for identifying patients who at particularly high adverse outcomes.Third, cut-off values of cys-C level in each study were different and we could not recommend appropriate cys-C level in clinical application. Fourth, publication bias test may be unreliable because the number of studies was less than the recommended arbitrary minimum number. Fifth, we did not use the continuous data to analyze the prognostic value of cys-C level due to limited studies reporting such data. Finally, generalizability of the current findings to younger HF patients should be with caution because this meta-analysis predominantly included older patients.

In conclusion, elevated circulating level of cys-C is possibly independently associated with an increased risk of all-cause mortality and rehospitalization in HF patients. Determination of cys-C level may improve classification of adverse outcomes in HF patients and help the clinician in therapeutic decision making.

## Supporting information

**Supplemental Table S1:** Sensitivity analyses on all-cause mortality

**Supplemental Table S2:** Sensitivity analyses on combination of mortality/rehospitalization

## Abbreviations

BMI: body mass index
BNP: B-type natriuretic peptide
CI: confidence interval
COPD: chronic obstructive pulmonary disease
cys-C/CYC: cystatin C
EF: ejection fraction
HF: heart failure
HFpEF: HF with preserved EF
HFrEF: HF with reduced EF
HR: hazard ratio
LVEF: left ventricular EF
NOS: Newcastle–Ottawa Scale
NP: not reported
NT-proBNP: N-terminal pro-BNP
SBP: systolic blood pressure
