# Supplementary material for: Cystatin C for predicting all-cause mortality and rehospitalization in patients with heart failure: a meta-analysis
Source: Biosci Rep. 2019 Feb 5;39(2):BSR20181761. doi: 10.1042/BSR20181761 (PMC6361773; doi:10.1042/BSR20181761)
Supplement: Supplementary file 1 [file bsr20181761_Supp1.pdf]

Supplemental Table S1 Sensitivity analyses on all-cause mortality

Removal of single study each time	Pooled hazard risk	95% confidence intervals	Heterogeneity across studies
Breidthardt et al. 2017 (28)	2.55	1.75–3.72	p=0.001; I^2 =74.0%
Campbell et al. 2009 (21)	2.39	1.65–3.47	p=0.001; I^2 =78.1%
Shlipak et al. 2005 (19)	2.39	1.63–3.50	p=0.001; I^2 =78.0%
Carrasco-Sánchez et al. 2014 (25)	2.19	1.56–3.08	p=0.001; I^2 =75.1%
Pérez-Calvo et al. 2012 (24)	2.28	1.58–3.30	p=0.001; I^2 =76.3%
Lassus et al.2007 (20)	2.23	1.56–3.19	p=0.001; I^2 =74.6%
Jackson et al. 2016 (27)	2.57	1.88–3.51	p=0.010; I^2 =62.3%
Ruan et al. 2014 (26)	2.30	1.58–3.30	p=0.001; I^2 =75.7%
Carrasco-Sánchez et al. 2011 (23)	2.17	1.57–3.01	p=0.001; I^2 =73.9%
