# Supplementary material for: Cystatin C for predicting all-cause mortality and rehospitalization in patients with heart failure: a meta-analysis
Source: Biosci Rep. 2019 Feb 5;39(2):BSR20181761. doi: 10.1042/BSR20181761 (PMC6361773; doi:10.1042/BSR20181761)
Supplement: Supplementary file 2 [file bsr20181761_Supp2.pdf]

Supplemental Table S2 Sensitivity analyses on combination of mortality/rehospitalization

Removal of single study each time	Pooled hazard risk	95% confidence intervals	Heterogeneity across studies
Manzano-Fernández et al. 2011 (22)	2.33	1.65–3.29	p=0.135; I^2 =55.3%
Campbell et al. 2009 (21)	2.14	1.53–3.01	p=0.070; I^2 =69.6%
Carrasco-Sánchez et al. 2011 (23)	1.83	1.36–2.46	p=0.703; I^2 =0.0%
